# Social Information Processing and Aggressive Behavior in Early Childhood: a Brazilian Birth Cohort Study

**DOI:** 10.1177/08862605251412358

**Published:** 2026-04-22

**Authors:** Luciana Rodrigues Perrone, Andreas Bauer, Suelen Cruz, Joseph Murray

**Affiliations:** a Postgraduate Program in Epidemiology, Federal University of Pelotas, Rua Marechal Deodoro, 1160, 3° Piso, CEP: 96020-220, Pelotas, Rio Grande do Sul, Brazil; b Human Development and Violence Research Centre (DOVE), Federal University of Pelotas, Rua Marechal Deodoro, 1160, 3° Piso, CEP: 96020-220, Pelotas, Rio Grande do Sul, Brazil

**Keywords:** child aggression, social information processing, SIP model, birth cohort, Brazil

## Abstract

**Background:**

Child aggressive behavior is normative in early childhood and generally declines through development. However, persistent aggression from ages 2-3 years is associated with social and health problems, including violence, into adulthood. An important theoretical model proposed by Crick and Dodge (1994) states that difficulties processing social information is implicated in persistent aggression from the preschool period onwards. This study aimed to investigate the influence of social information processing (SIP) in early childhood in a large Brazilian birth cohort, accounting for other social, family, and child factors.

**Method:**

We used data on 3532 children in a population-based birth cohort study in Pelotas City, Brazil. SIP was measured at age 4 years using the SIPI-P instrument – a structured interview with children – from which three variables were derived: hostile attribution bias, aggressive response generation, and a competent assessment score. Aggressive behavior was measured using the ELDEQ questionnaire completed by mothers at age 6-7. A range of child, mother, family, and community factors were measured as possible confounders between birth and age 4 years.

**Results:**

In crude analyses, both aggressive response generation and competent assessment, but not hostile intent attribution, were associated with increases in later child aggressive behavior. The relationship between SIP and child aggression was confounded by multiple environmental factors such as community violence, maternal education, family income, parental antisocial behavior, maternal hostile attribution bias, maternal depression, and coercive parenting, as well as child factors such as sex and language development. After adjusting for confounders, only aggressive response generation remained associated with later child aggression.

**Conclusions:**

In this Brazilian context, aggressive response generation was the only significant aspect of SIP longitudinally associated with child aggression. Other psychosocial factors showing strong associations with child aggression in this setting require further research to identify the most appropriate targets for early preventive interventions.

## Introduction

Longitudinal studies following children from infancy show that physical aggression emerges during the first 2 years of life and peaks in frequency between 2 and 3 years, before generally declining through development (Côté et al., 2007; Nærde et al., 2014). During early childhood, most children can be observed, at some point, hitting, biting, or kicking other children or even an adult (Tremblay, 2004). Despite a general decline in this behavior, a small proportion of children tend to maintain elevated levels of physical aggression throughout childhood, which is associated with continuity in aggression into later life (Teymoori et al., 2018). This small group of children with more persistent aggression tend to be boys with impulsive and hyperactivity difficulties, and low school performance (Barker et al., 2008). A trajectory of persistent physical aggression is associated with a range of later life problems, such as more serious violence in adulthood, difficulty in social adaptation, and abuse of alcohol and illicit drugs (Barker et al., 2008). Identification of early risk factors predicting elevated aggression in childhood beyond the initial peak at age 2 years is therefore important to prevent later violence and other social problems (Barker et al., 2008).

In a classic theory, Crick and Dodge (1994) proposed that child aggressive behavior can be understood as arising from atypical social information processing (SIP). Specifically, they propose that five steps of cognitive processing of information from a social interaction occur before an action is taken in a sixth step - according to the response judged most appropriate to the situation. The six steps of SIP are: (1) encoding social cues; (2) interpretation of cues; (3) clarification of goals; (4) response access or construction; (5) response decision and (6) behavioral enactment. According to this model, more aggressive children tend to have lower competencies in processing social information through the five cognitive steps of the SIP process (Ziv, 2012). Steps two, four and five are the most studied in the literature (Runions & Keating, 2007). In step two a child may interpret cues with hostile intent attribution, which is defined as an over-attribution of hostility to other people’s behavior, even in ambiguous situations (Choe et al., 2013; Godleski & Ostrov, 2020). It is hypothesized that hostile intent attribution increases the likelihood of an aggressive response by the child. Steps four and five in the SIP model concern behavioral response planning. Crick and Dodge (1994) proposed that children with aggressive behavior consider fewer potential responses to social situations, which tend to be more aggressive and less prosocial. They also suggested that, in step five, more aggressive children tend to evaluate aggressive responses more favorably (in terms of social consequences) and evaluate competent responses more negatively. In the SIP model, each of the six steps is affected by the child’s memory of past situations, learned social rules, social schemas, and knowledge of appropriate or inappropriate social behaviors (Ziv, 2012).

The current literature highlights key risk factors for child aggression across multiple domains. For example, negative temperament (Vitaro et al., 2006) and low IQ (Huesmann et al., 1987) have been identified as individual characteristics that may increase aggressive behavior. Previous studies also emphasise low socioeconomic status as one of the most consistent predictors of child behavior problems (Song et al., 2020). An underlying mechanism linking sociodemographic factors and problematic child behaviors is stress in the family environment, which impacts levels of parental involvement, as well as types of parenting practices and interactions with children that affect their development (Ziv & Sorongon, 2011). In particular, in stressful family environments, harsh and inconsistent parenting practices and psychological control are understood to reinforce child aggression (Godleski & Ostrov, 2020). On the other hand, a strong child-parent attachment has been associated with reduced levels of young children’s aggression (Juan et al., 2020). Exposure to violence, maternal depression, and maternal hostile intent attribution are other critical factors cited in the literature that may alter child SIP and aggressive behavior (Godleski & Ostrov, 2020; Schultz et al., 2000; Ziv, 2012).

Although SIP is a powerful theoretical framework for understanding child aggression, little is known about its role for children of preschool age (Denham et al., 2002; Ziv, 2012). Examining these processes in young children, before longer-term interaction styles are fully developed and behavioral problems become more entrenched, is an important area for further research to help guide preventive intervention (Mayeux & Cillessen, 2003; Perhamus & Ostrov, 2020). Denham et al. (2002) emphasize that children’s learning about emotions and social competence skills should not be left to chance. As the SIP model describes specific steps where competent processes could be taught through practice and demonstration, preschoolers showing difficulties could be supported and potentially thereby reduce life-long conflicts based on atypical interpretations of social cues (Ziv, 2012). Currently, the evidence base is scarce on the validity of the SIP model for preschool children, although some studies have suggested that problems in several SIP steps, particularly hostile intent attribution, hypothetical response generation, and response assessment are associated with early externalizing behaviors (Lansford et al., 2006; Ziv & Sorongon, 2011). Importantly, recent longitudinal findings have reinforced the long-term implications of SIP. For instance, Dodge (2024) demonstrated that defensive mindsets – that is, enduring cognitive-emotional orientations characterized by heightened vigilance to threat and expectations of hostility – underlying SIP patterns developed in early childhood significantly predict externalizing psychopathology, incarceration, and economic and relational dysfunction well into adulthood, supporting the idea of developmental cascades initiated by early cognitive-social biases. Interestingly, Ziv (2012) also demonstrated a mediating role of SIP between exposure to violence and both externalizing and internalizing behaviors in preschool children.

As well as a need for further evidence on the role of social information processing on aggression for preschool children, research is dominated by studies in high-income countries, and there is little evidence across the global south, which has enormous variations in social, economic, and cultural conditions. Understanding the roots of aggression and violence in low- and middle-income countries with high rates of violence is particularly important to achieve significant reductions of these problems across the globe. Brazil, a middle-income country, where the current study was conducted, has seen an enormous rise in serious violence in the last three decades and currently has the highest number of homicides in any country worldwide (Institute for Health Metrics and Evaluation, 2022). Links between child behavior problems and later violence have been demonstrated in this setting (Murray et al., 2015). However, the social determinants of early social understanding (such as in the SIP model) and their role in understanding child aggression in this setting have not been clarified. Thus, this study aimed to investigate the influence of social information processing on aggression in young children in a large Brazilian cohort. Our hypotheses are: 1) children with less competent SIP will present higher aggression in crude analyses; 2) considering the social context of Brazil with high rates of violence and other important social determinants of behavior, after adjusting for social, family, and child factors as confounders of the association, effect sizes between SIP and child aggression will decrease, but remain significant.

## Methods

### Study Design and Participants

The 2015 Pelotas Birth Cohort is an ongoing, population-based, prospective longitudinal study investigating a variety of determinants of health and social outcomes. In 2015, all maternity hospitals in Pelotas City, Southern Brazil, were visited daily, and all children born to women who lived in the urban area of the city were eligible to participate in the cohort (Hallal et al., 2018; Murray et al., 2024). Of 4275 participants enrolled at birth (98.7% response rate), 4018, 4010, and 3867 were assessed at ages 2, 4, and 6-7 years, respectively. Of those, 3532 had valid data on both SIP at 4-years and child aggression at 6-7-years follow-ups and were included in the current analyses. RedCap was used for data collection (Harris et al., 2009).

The general cohort study protocol was approved by the Ethics Committee of the Physical Education School of Federal University of Pelotas (26746414.5.0000.5313). The Ethics Committee of Medical School of Federal University of Pelotas approved the 4-year (03837318.6.0000.5317) and 6-7-year (51789921.1.0000.5317) follow-up protocols. Written consent was obtained from carers for all interviews and participants were free to stop assessments at any time.

### Child Social Information Processing - SIP

Social information processing was measured at age 4 years using the Social Information Processing Interview – Preschool Version (SIPI-P) (Ziv & Sorongon, 2011). The structured interview is based on illustrated stories about cub bears, which are told to the child. Children are asked questions about what is happening in the story and their responses are used to measure three social processing variables: hostile intent attribution, aggressive response generation, and a competent assessment score. Although previous studies with up to a few hundred participants have used multiple stories to evaluate SIP, considering the large sample size of this Brazilian birth cohort study, it was only possible to implement SIPI-P using one story vignette.

The story used in this study depicts an ambiguous social situation where two bears are playing together with blocks when a third person – Maria – arrives and asks if she can join the game. One of the bears denies the request, saying the teacher told them only two can play with the blocks. At this moment, the story is paused, and the interviewer asks the child whether they think the bear that did not let Maria play is mean or not mean. A reply of “mean” is classified as indicating a hostile intent attribution in this ambiguous situation. This represents the first dichotomous SIP exposure variable.

Next, the interviewer asks the child an open-ended question: “What would you do if this [excluded from the game] happened to you?”, and the child’s verbatim answer was recorded. Responses were subsequently classified as aggressive or non-aggressive responses, producing a dichotomous variable called “aggressive response generation”. Any response indicating an intention of hurting who had excluded the child (e.g., “I’ll hit them”) or ending the game for the other children (e.g., “I’ll kick the blocks”) was coded as an aggressive response generation option and otherwise was coded as non-aggressive.

Finally, a “competent assessment score” was created referring to how the child evaluated different possible responses to the situation. The interviewer provided three hypothetical responses that Maria might show (competent, aggressive, and inept). The “competent” response described Maria asking to play later. The “aggressive” response described Maria messing up the blocks. The “inept” response described Maria crying. After each response description, the child was asked: 1) “Is what Maria said/did a good thing or a bad thing to say/do?”, 2) “If you did that, do you think the other children would like you?”, 3) “Do you think the other children would let you play if you did that?”. A total “competent response evaluation” score was derived as follows. First, for the description of Maria responding competently, a score of 1 was given to each positive answer the child gave to the three questions. For the description of Maria responding either with aggression or with an inept response, scores of 1 were given to negative answers to the three questions. Individual dichotomous scores were then summed from the nine questions to form a total score ranging from 0 to 9. A minimum of six valid answers were required, and otherwise, the variable was coded as missing (children sometimes failed to answer or gave non-sensical answers). For children with valid answers for six to eight questions, scores were prorated.

### Child Aggressive Behavior

Aggressive behavior was measured at age 6-7 years, using a questionnaire from a Canadian study entitled *Etude Longitudinale du Development des Enfants du Quebec* – ELDEQ (Orri et al., 2021; Tremblay, 2004). There are 14 aggression items in total, and carers are asked to report on the frequency with which their child showed each behavior in the last three months, ranging from “never” (0) to “often” (2). Factor analysis of the questionnaire in Pelotas showed that only nine items were suitable for forming a scale for that sample. These include: 1) Hit, bit, or kicked others; 2) Fought with others; 3) Bullied, provoked or annoyed others; 4) Reacted aggressively when provoked; 5) Tried to dominate another child; 6) Reacted aggressively when contradicted; 7) Reacted with anger or fought when someone hit him/her accidentally; 8) Physically attacked other people; 9) Reacted aggressively when something was taken away from him/her. Therefore, the final sum score of physical and reactive aggressive behavior, based on 9 items, ranges from 0 to 18 points.

### Confounding variables

The following confounding variables were measured in the perinatal assessment: child sex (female vs. male), maternal age in years (categorized as <20, 20-34, ≥35), maternal education in completed years (categorized as 0-4, 5-8, 9-11, ≥12) and family income in quintiles (originally collected in minimum wages). At age 2 years, the coercive parenting scale (scores from 0 to 15) was obtained from a five-item subscale of the Parent and Family Adjustment Scales (PAFAS) (Sanders et al., 2014).

At age 4-years, the following community and family confounders were assessed: neighborhood violence (scores from 0 to 12), measured using a 4-item instrument proposed by Mujahid et al. (2007), maltreatment (scores from 0 to 4) obtained from maltreatment module questions of the Juvenile Victimization Questionnaire (JVQ-R2) (Finkelhor et al., 2005), mother with partner (yes vs. no), maternal depression (score from 0 to 30) measure using the Edinburgh Postnatal Depression Scale (EPDS) (Cox et al., 1987), parents’ antisocial behavior (scores from 0 to 12) resulting from the sum of six dichotomous questions posed to the mother about behaviors of each parent after age 15 years, on the Mini International Neuropsychiatric Interview (M.I.N.I.) (Amorim, 2000; de Azevedo Marques & Zuardi, 2008), and maternal hostile intent attribution (0 to 25) measured using the Parental Hostile Attribution Questionnaire (parental HAQ) (Halligan et al., 2007).

Finally, at the individual level, language (score of 0 to 133) was scored using the sum of two instruments - expressive language (100 items) assessed by the USP Expressive Vocabulary Test (TVexp-100) and receptive language (33 items) assessed by the USP Auditory Vocabulary Test (TVAud-33) (Capovilla et al., 2011). More details about the measurement of psychological instruments used as confounding variables are shown in the supplementary material.

### Statistical analysis

The analyses proceeded in three stages. First, children included in the current study and those excluded were compared on sociodemographic variables at baseline using the chi-square test.

Second, descriptive statistics (% and means/standard deviations) were calculated for the three exposure variables of the SIP model, and the aggression outcome variable, both for the whole analytic sample and stratified according to confounding variables. Confounding variables scores were dichotomized or divided into terciles for these descriptive analyses. For the first two exposures (hostile intent attribution and aggressive response generation) chi-square tests were used to compare across categories of confounding variables. For the third exposure (competent assessment score) and the outcome (aggressive behaviors score) Student’s t-test was used for dichotomous confounders and ANOVA for confounders with three or more categories.

Finally, due to the count nature of the outcome, multivariate Poisson regression with robust variance was used to estimate crude and adjusted relative risks between the three SIP exposure variables and the outcome child aggression variable. As each SIP exposure variable was examined separately, different sample sizes were used in each analysis. The crude regression analyses were performed with the same samples as the adjusted analyses (requiring valid data on confounders). In these regression models, confounder scores were used in their original format. For all analysis, a significant level of 5% was used. All analyses were performed in STATA 15.1.

## Results

**Figure 1:**
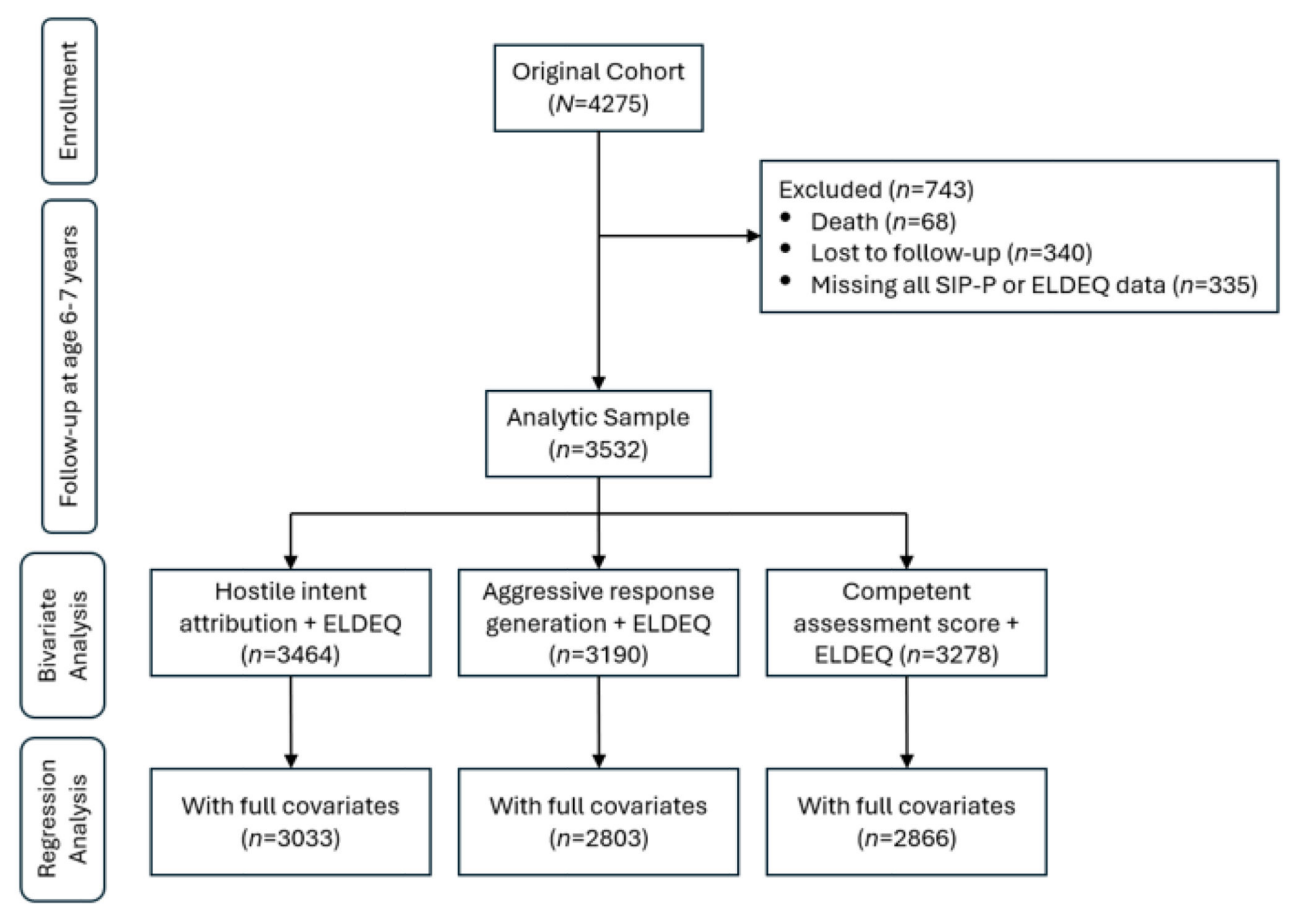
Flow diagram of included/excluded sample children.

Of the 4275 children in the 2015 Pelotas Birth Cohort, 743 were excluded from the present study because of death (n=68), loss to follow-up at age 6-7 years (n=340), or not having valid data on SIP or the ELDEQ aggression questionnaire (n=335) (Figure 1). Table 1 shows characteristics of the original cohort, as well as the sample of 3532 participants included in the analysis and those excluded. Half (50.2%) of the children in the analytic sample were female; most mothers were 20-34 years old (71.0%) and had nine or more years of schooling (65.4%). Children excluded from the study had generally similar characteristics. However, with the large sample size, there were statistically significant small differences in child sex, maternal education, and family income, compared to the analytic sample (Table 1).

Considering the three SIP exposure variables, 70.5% of children (n = 2442) showed hostile intent attribution and 12.9% generated aggressive hypothetical responses (n = 410) in the SIPI-P task. The mean (sd) of the SIP competent assessment score was 5.87 (1.92). The mean (sd) of the aggressive behavior outcome score was 3.28 (3.30).

Table 2 shows descriptive statistics for the three SIP exposure variables and the outcome aggressive behavior score, according to categories of the confounders. Neighborhood violence was associated with lower competent assessment scores and aggressive behavior scores. Considering sociodemographic factors, boys were more likely to generate SIP-aggressive responses than girls. Boys also had lower SIP competent assessment scores and higher aggressive behavior outcome scores on average. There was no evidence of a sex difference in the prevalence of hostile intent attribution. Maternal education and family income were strongly associated with all three SIP exposure variables and the aggression outcome score. More precisely, children with more educated mothers and higher family incomes had a lower prevalence of hostile intent attribution and aggressive response generation. They also had higher competent assessment scores and lower aggressive behavior outcome scores. Finally, children of younger mothers had a higher prevalence of hostile intent attribution and had lower mean competent assessment scores.

Among family-level variables, hostile intent attribution was strongly associated with parental antisocial behavior and with maternal hostile intent attribution, but no other family-level variable. Aggressive response generation was more prevalent in children of depressed mothers and children whose mothers showed higher hostile intent attribution. Children’s competent assessment score was significantly associated with four family-level variables (higher scores for children whose mothers had partners, mothers without depression, lower parental antisocial behavior, and less maternal hostile intent attribution. Finally, child aggressive behavior was associated with all variables at the family level — higher scores associated with coercive parenting, maltreatment, mother with no partner, maternal depression, parental antisocial behavior, and maternal hostile intent attribution.

Considering the individual level, language was not associated with child hostile intent attribution. However, children with higher language scores were less likely to generate SIP aggressive responses, had higher competent evaluation scores, and lower aggressive behavior scores.

Finally, associations between the three SIP exposures and childhood aggressive behavior were examined in multivariate regression. Table 3 shows crude and adjusted results for the aggressive behavior score, for each exposure. In crude analysis, children who exhibited hostile intent attribution scored, on average, 3% higher in aggressive behavior compared to their peers who did not demonstrate this attribution; however, this difference was not statistically significant (p = 0.429). Children who generated an aggressive response in the SIP task scored 18% higher in the outcome on average when compared to those without aggressive response generation (p = 0.002). Furthermore, for each additional unit increase in the competent assessment score, children exhibited, on average, a 3% reduction in aggressive behavior outcomes (p = 0.002).

After controlling for confounding variables, only the aggressive response generation variable maintained significant, albeit slightly attenuated Specifically, the predictive values were 0.98 (p=0.688) for hostile intent attribution, 1.13 (p=0.017) for the generation of aggressive responses in the SIP task, and 0.99 (p=0.446) for the competent assessment score.

## Discussion

This current study investigated longitudinal associations between three aspects of children’s social information processing at the age of 4 years and their aggressive behavior at 6-7 years, in a Brazilian birth cohort, representing one of the largest, population-based studies on the topic worldwide. Notably, many low-and middle-income countries have high rates of violence, including in Brazil where the current study was set. If determinants of child aggression can be identified in such settings, this could support early interventions to prevent violence later in the life-course. Findings from the current study generally confirmed expectations about the community, sociodemographic, family conditions and child factors associated with children showing problematic SIP (hostile intent attribution, more hypothetical aggressive responses, worse competent assessment scores), and more aggressive behavior. However, our findings suggest that SIP difficulties at age 4 played only a small part in understanding aggressive behavior among children at age of school-entry.

Only aggressive response generation in the SIP model remained significantly associated with later aggressive behavior in the current study, indicating that having a limited repertoire of competent social responses may play a particularly important role in the persistence of aggressive behavior in early childhood. This is consistent with recent evidence from a trial study by Alsem et al. (2023), which found that a cognitive-behavioral intervention incorporating immersive virtual reality was effective in decreasing aggressive behavior in children, especially by improving children’s ability to generate competent social responses. While these findings highlight the importance of response generation, other studies have identified different SIP components as more relevant. For example, in a study conducted in the United States with 196 children aged 48 to 61 months, Ziv and Sorongon (2011) found that only competent assessment score was associated with aggression, after controlling for sex, race, and language skills. In our current study, an association was also found in crude analysis after adjusting for confounding variables. The fact that we used a broader set of covariates may explain, in part, the differences in findings. In the study by Ziv and Sorongon (2011), there was not an association between hostile intent attribution and aggression, which aligns with our study. They also had null results for aggressive response generation, which diverged from our findings. It is important to note that, despite similar measures of SIP, Ziv and Sorongon (2011) categorized response generation into three categories (inept, competent, and aggressive) instead of two, as we did in our study (aggressive and non-aggressive), which may also account for the differences in results.

The bivariate analyses concerning covariates in our study showed multiple individual, family, and community factors related to both SIP and child aggression. Although various previous studies reported significant associations between SIP and child aggression (Lansford et al., 2006; Schultz et al., 2010), they included none or a relatively small number of potential confounders (e.g., only child sex and family income). For example, in a study of 193 4-5 year-old children in Germany, two SIP variables (aggressive response generation and evaluation of aggressive responses) were associated with child aggression, but no confounders were adjusted for (Helmsen et al., 2012). Given the many child, family, and community variables relevant to both SIP and child aggression assessed in the current study, prior studies may have overestimated the effects of SIP on early childhood aggression because few or no confounders were considered.

Another possible explanation for the lack of influence of some SIP variables on child aggression in the current study concerns the social context of Brazil, and the possibility that factors such as high levels of social disadvantage and exposure to violence are overwhelming influences on child behavior - swamping the relevance of more subtle SIP factors. A recent meta-analysis, (Verhoef et al., 2019) found a positive association between hostile intent attribution and child aggression, but effect sizes between the studies varied significantly. This might be explained by methodological differences, but also unexplored socio-cultural differences that could potentially moderate such effects. A relevant more general hypothesis that individual-level factors are less important for aggression in contexts of greater social adversity has been called the “social push” hypothesis by Raine (2013).

It is important to point out that the literature on SIP and child behavior does not have uniform results across studies, and this appears partly because of the different ways that SIP constructs are operationalized and categorized, as well as different analytic approaches. For example, while Lansford et al. (2006) considered SIP response generation and SIP response evaluation as separate variables, Ziv and Arbel (2021) combined them, creating a single score. While both these studies focused on whether children’s responses to SIP vignettes were aggressive versus not aggressive, others have classified the responses as competent vs. non-competent (Ziv & Sorongon, 2011).

Hostile intent attribution is the aspect of SIP most studied concerning aggressive behavior. Just over two-thirds of the 4-year-old children in the current study showed hostile intent attribution, which might be expected given their young age. According to Dodge (2006), hostile intent attribution about others’ intentions is universally acquired in early childhood and then, except for a small proportion of children, gradually replaced through development by a benign attributional style. This could explain why children who exhibited a hostile intent attribution at age four did not have higher scores of aggressive behaviors at 6-7 years. It is also possible that the high rate of hostile attribution in the current study was partly a function of used a closed question about whether or not the character was mean, which might have elicited more “mean” attributions, than an open-ended question about the character, as has sometimes been used in prior research.

We observed several factors differentiating most children with hostile intent attribution from the remainder. First, in line with other studies suggesting an intergenerational transmission of hostile intent attribution (Godleski & Ostrov, 2020), we found a significant association between maternal hostile intent attribution and child hostile intent attribution. A UK prospective study (Healy et al., 2015) also found an association between maternal hostile intent attribution at child age 18 months and child hostile intent attribution at age 5 years, but there was no cross-sectional relationship between these variables at age 5 years. According to the authors, the longitudinal nature of the association supports the assumption that children observe and internalize their parents’ hostile interpretive style over time. The study by Healy et al. (2015) also found a direct association between maternal hostile intent attribution and child aggressiveness, in line with our findings.

However, not all studies report an association between maternal and child hostile attribution style. In another UK sample of 5-7 year old children, Halligan et al. (2007) found that while maternal hostile intent attribution was associated with child externalizing problems, it was not related to child hostile intent attribution — suggesting other possible mechanisms for the link with externalizing, such as parents with hostile attribution styles being more likely to use harsh parenting practices that are related to child problem behavior (Ziv & Arbel, 2021).

In the current study, there was an association between parental antisocial behavior and child hostile intent attribution, as well as lower child competent assessment scores, and more child aggression, indicating both intergenerational transmission of antisocial behavior, which is well documented in numerous studies (Besemer et al., 2017), and possible influences of parental antisocial behavior on child SIP. Notably, low maternal education and low family income are also associated with all aspects of SIP, as well as child aggression. Low education and income represent broad markers of adversity and many possible mechanisms in the family or community environments that could influence child outcomes.

As expected, girls showed more competent social information processing (Lansford et al., 2006) and less aggressive behavior than boys (Denham et al., 2002). However, this was not the case for hostile intent attribution specifically, which was similarly highly prevalent across both sexes. Considering that boys tend to be more involved in physical aggression while girls are more likely to be involved in relational aggression (Helmsen et al., 2012), we do not discard the possibility that higher levels of aggression among boys in the current study relate to young child age and physical aggression being more easily perceived by mothers, who reported on the outcome.

Children who lived in more violent neighborhoods in Pelotas City were characterized by lower competent assessment scores in the SIP task (but not more hostile intent attribution or aggressive response generation), in line with previous studies (e.g., Ziv, 2012). However, we did not find an association between child maltreatment and SIP variables, in contrast to previous research (Johnson et al., 2020). Although the maltreatment measure used in the current study is associated with numerous other factors in this cohort (Buffarini et al., 2021, 2022) and with child aggression in the current study, providing some criterion validity for the measure, it is possible that a relatively low prevalence of maltreatment up to age four years meant the study lacked power to capture significant smaller associations.

The main strength of this study is the large population-based sample in Brazil, providing estimates of the longitudinal association between SIP and childhood aggression in an understudied social and cultural context. The study used a wide range of measures to consider factors that might covary with SIP and child aggression, and adjust for them in analyses, and had a high follow-up rate for such a large study. However, the findings need to be interpreted in the context of limitations. First, due to the scale of this large epidemiological study, we utilized one story vignette in the SIP assessment. While this approach effectively captured key information, as evidenced by numerous bivariate associations with other variables, it may have resulted in lower measurement quality compared to using multiple vignettes, potentially diminishing the estimated effect sizes. Second, although response rates were high, we observed minor differences in baseline characteristics between the analytic sample and those excluded due to missing data, which could introduce bias into the results. Third, although we controlled for several important confounding factors, some were not considered in the analyses. The lack of data on child IQ and temperament, for example, is highlighted as a limitation of the study. Finally, while we generally characterized the family and social context well, like most previous research, our focus was primarily on the mother-child relationship, omitting an assessment of the father’s role in child SIP and aggression.

While our sample included families from a single Brazilian city, it comprised participants from a range of socioeconomic and demographic backgrounds. Therefore, although the findings may not fully represent all populations, they are likely to be informative for other Brazilian contexts. Although potential sex differences were explored, future studies should investigate how other dimensions of diversity – such as ethnicity, neurodiversity, and family composition – may influence the development of SIP patterns and aggressive behavior. This is particularly relevant comparing across socio-cultural contexts, where parenting norms and social-cognitive processing may vary significantly. As research expands, incorporating intersectional approaches will be essential to better understand how multiple forms of diversity intersect to shape child development.

## Conclusion

In conclusion, considering three important aspects of child SIP, only aggressive response generation at 4-years significantly predicted aggressive behavior at 6-7-years. Child aggression is undoubtedly a highly complex behavior, with multiple bio-psycho-social determinants. The findings of this study suggest that in this Brazilian context, other psychosocial determinants are likely very important in the development and persistence of child aggression. Family and community contexts associated with elevated child aggression at age of school entry require further research in this context to identify the most salient determinants to target in early preventive interventions.

## Supplementary Material

Supplementary Files

## Figures and Tables

**Table 1:** Sociodemographic characteristics at birth of the original cohort, and comparisons between children in the analytic sample, and those excluded from the analyses. 2015 Pelotas Birth Cohort.

	Original cohort *(n=4275)* N (%)	Included *(n=3532)* N (%)		Excluded *(n=743)* N (%)
Child sex			***p* = 0.013**	
Male	2164 (50.6)	1757 (49.8)		407 (54.8)
Female	2111 (49.4)	1775 (50.2)		336 (45.2)
Maternal age^a^			*p* = 0.149	
<20 yrs	623 (14.6)	519 (14.7)		104 (14.0)
20-34 yrs	3018 (70.6)	2507 (71.0)		511 (68.9)
≥35 yrs	633 (14.8)	506 (14.3)		127 (17.1)
Maternal schooling^a^			***p* = 0.013**	
0-4 yrs	391 (09.2)	320 (09.1)		71 (09.6)
5-8 yrs	1095 (25.6)	899 (25.5)		196 (26.4)
9-11 yrs	1458 (34.1)	1241 (35.1)		217 (29.2)
≥12 yrs	1330 (31.1)	1071 (30.3)		259 (34.8)
Family income^b^			***p* = 0.001**	
Quintile 1	846 (19.8)	695 (19.7)		151 (20.3)
Quintile 2	859 (20.1)	724 (20.5)		135 (18.2)
Quintile 3	853 (20.0)	729 (20.7)		124 (16.7)
Quintile 4	856 (20.0)	711 (20.1)		145 (19.5)
Quintile 5	859 (20.1)	671 (19.0)		188 (25.3)

a 1 missing,

b 2 missing.

* p values from Chi-square test comparing included and excluded children.

**Table 2:** Description of SIP variables (hostile intent attribution, aggressive response generation, and competent assessment scores) at age 4 years and aggressive behavior at age 6-7years among children in the 2015 Pelotas Birth Cohort, according to community, sociodemographic, and family factors, and child language.

	N(%)	Hostile intent attribution (%) *n=3464*	Aggressive response generation (%) *n=3190*	Competent assessment score mean (sd) *n=3278*	Aggressive behavior score mean (sd) *n=3532*
**Total**		*70.5*	*12.9*	*5.87 (1.92)*	*3.28 (3.30)*
**Community factor**					
**Neighborhood violence** ^a^		*p=0.082*	*p*=0.052	***p*=0.0001**	***p*<0.0001**
Tercile 1	1430 (40.7)	68.6	11.5	6.03 (1.93)	2.89 (3.13)
Tercile 2	1201 (34.1)	71.5	12.9	5.85 (1.92)	3.31 (3.17)
Tercile 3	885 (25.2)	72.7	15.2	5.67 (1.85)	3.85 (3.60)
**Sociodemographic factors**					
**Child sex**		*p*=0.670	***p*=0.025**	***p*=0.0006**	***p*<0.0001**
Male	1757 (49.8)	70.2	14.2	5.76 (1.90)	3.61 (3.39)
Female	1775 (50.2)	70.8	11.6	5.99 (1.92)	2.96 (3.18)
**Maternal age**		***p*=0.009**	*p*=0.200	***p*=0.0001**	*p*=0.0667
<20 yrs	519 (14.7)	76.2	14.6	5.55 (1.94)	3.57 (3.54)
20-34 yrs	2507 (71.0)	69.5	12.9	5.91 (1.92)	3.25 (3.29)
≥35 yrs	506 (14.3)	69.7	10.7	6.05 (1.82)	3.12 (3.12)
**Maternal education** ^b^		***p*<0.001**	***p*=0.016**	***p*<0.0001**	***p*<0.0001**
0-4 yrs	320 (09.1)	70.1	15.0	5.33 (1.79)	4.13 (4.02)
5-8 yrs	899 (25.5)	75.6	15.3	5.41 (1.84)	3.57 (3.44)
9-11 yrs	1241 (35.1)	71.7	12.7	5.84 (1.89)	3.14 (3.19)
≥12 yrs	1071 (30.3)	64.9	10.5	6.46 (1.88)	2.95 (2.98)
**Family income** ^c^		***p*<0.001**	***p*=0.001**	***p*<0.0001**	***p*=0.0001**
Quintile 1	695 (19.7)	73.2	16.0	5.47 (1.89)	3.74 (3.63)
Quintile 2	724 (20.5)	73.0	13.2	5.55 (1.86)	3.38 (3.32)
Quintile 3	729 (20.7)	71.9	15.3	5.92 (1.88)	3.21 (3.33)
Quintile 4	711 (20.1)	70.3	10.6	6.04 (1.91)	3.14 (3.18)
Quintile 5	671 (19.0)	63.6	09.4	6.40 (1.89)	2.93 (2.93)
**Family factors**					
**Coercive parenting** ^d^		*p*=0.248	*p*=0.116	*p*=0.1545	***p*<0.0001**
Tercile 1	1410 (43.2)	69.2	12.0	5.90 (1.94)	2.67 (2.94)
Tercile 2	1032 (31.6)	71.3	14.7	5.95 (1.91)	3.48 (3.26)
Tercile 3	824 (25.2)	72.4	12.0	5.77 (1.90)	4.13 (3.69)
**Maltreatment** ^e^		*p*=0.357	*p*=0.757	*p*=0.9750	***p*<0.0001**
None	3124 (88.6)	70.3	12.9	5.88 (1.91)	3.15 (3.24)
Any	401 (11.4)	72.5	12.4	5.87 (1.93)	4.26 (3.59)
**Mother with partner** ^f^		*p*=0.185	*p*=0.480	***p*=0.0002**	***p*<0.0001**
No	692 (19.7)	72.6	13.7	5.63 (1.89)	3.78 (3.64)
Yes	2829 (80.3)	70.0	12.7	5.94 (1.91)	3.16 (3.20)
**Mother depression** ^f^		*p=0.057*	***p*=0.023**	***p*=0.0350**	***p*<0.0001**
No	2596 (73.7)	69.6	12.1	5.92 (1.93)	3.00 (3.11)
Yes	925 (26.3)	73.0	15.1	5.76 (1.88)	4.07 (3.62)
**Parents antisocial behavior** ^g^		***p*=0.001**	*p*=0.456	***p*=0.0173**	***p*<0.0001**
None	2353 (69.4)	68.8	12.4	5.94 (1.90)	2.90 (3.06)
Any	1038 (30.6)	74.7	13.4	5.77 (1.93)	3.98 (3.52)
**Maternal HIA** ^h^		***p*=0.005**	***p*=0.037**	***p*<0.0001**	***p*=0.0004**
Tercile 1	1189 (33.9)	68.6	11.8	6.14 (1.89)	3.04 (3.28)
Tercile 2	1273 (36.3)	69.5	11.8	5.82 (1.88)	3.22 (3.17)
Tercile 3	1048 (29.8)	74.5	15.2	5.65 (1.94)	3.59 (3.40)
**Child**					
**Language** ^i^		*p*=0.649	***p*<0.001**	***p*<0.0001**	***p*<0.0001**
Tercile 1	1168 (33.6)	71.4	15.7	5.14 (1.72)	3.69 (3.50)
Tercile 2	1205 (34.7)	70.1	14.1	5.79 (1.90)	3.33 (3.38)
Tercile 3	1100 (31.7)	69.7	09.3	6.68 (1.79)	2.79 (2.90)

a 16 missings,

b 1 missing,

c 2 missings,

d 266 missings,

e 7 missings,

f 11 missings,

g 141 missings,

h 22 missings,

i 59 missings in the analytical sample.

HIA = hostile intent attribution. For hostile intent attribution and aggressive response generation, *p* values are from Chi-Square tests. For competent assessment score and aggression behavior score, t-tests were used for dichotomous exposures and ANOVA for ordinal variables.

**Table 3:** Crude and adjusted risk ratios for aggressive behavior regressed on SIP variables. 2015 Pelotas Birth Cohort.

	Aggressive behavior score					
	Crude analysis	95% CI	*p*-value	Adjusted analysis	95% CI	*p*-value
**Hostile intent attribution** *(n=3033)*						
No	1.00			1.00		
Yes	1.03	(0.95, 1.12)	0.429	0.98	(0.91, 1.06)	0.688
**Aggressive response generation** *(n=2803)*						
No	1.00			1.00		
Yes	1.18	(1.06, 1.32)	**0.002**	1.13	(1.02, 1.25)	**0.017**
**Competent assessment score** *(n=2866)*	0.97	(0.95, 0.99)	**0.002**	0.99	(0.97, 1.01)	0.446

* Poisson Regression. Adjusted for neighborhood violence, child sex, maternal age, maternal schooling, family income, maltreatment, mother with partner, maternal depression, parents’ antisocial behavior, coercive parenting, maternal hostile intent attribution, and language.
