# Supplementary material for: Social Information Processing and Aggressive Behavior in Early Childhood: a Brazilian Birth Cohort Study
Source: J Interpers Violence. Author manuscript; Available in PMC 2026 Aug 14. (PMC7619365; doi:10.1177/08862605251412358)
Supplement: Supplementary file 1 [file EMS213946-supplement-Supplementary_Files.docx]

**Social Information Processing and Preschool Children’s Aggressive Behavior in a Brazilian Birth Cohort Study**

**SUPPLEMENTARY MATERIAL**

**DETAILS OF MEASURES OF CONFOUNDING VARIABLES**

**Neighborhood Violence**

The variable neighborhood violence was measured by asking mothers four questions about fights with weapons, gang fights, sexual assault or rape, and robbery or mugging in the neighborhood, during the past 6 months (MUJAHID *et al.*, 2007). Each answer ranges from “often” (3) to “never” (0), resulting in a score ranging from 0 to 12. The higher the score, the higher the neighborhood violence.

**Child Maltreatment**

Child maltreatment was measured by the Juvenile Victimization Questionnaire, 2^nd^ edition, Screener Sum Version, Caregiver Lifetime Form (JVQ-R2) (FINKELHOR *et al.*, 2005) answered by mothers. The maltreatment module consists of 4 dichotomous items asking if the child ever experienced physical abuse, emotional abuse, neglect, or had been kept away from a parent by the other parent (yes vs. no). The four items were summed to form a score ranging from 0 to 4.

**Maternal Depression**

Maternal depression was measured by the Edinburgh Postnatal Depression Scale (EPDS). This scale indicates the intensity of depressive symptoms over the preceding seven days. It is a screening, self-administered scale, with 10-items each having four possible responses, ranging from 0 to 3. Questions were read to mothers by a trained interviewer, given literacy issues. Although the original instrument is a postnatal depression scale, it was validated to measure maternal depression at other times (SANTOS *et al.*, 2007). The 10 items are summed, resulting in a total score ranging from 0 to 30, with higher scores indicating higher levels of maternal depression.

**Parental Antisocial Behavior**

Parental antisocial behavior was measured using the Mini International Neuropsychiatric Interview (M.I.N.I.) – version 5.0 (Antisocial Personality Module – current behaviors) (AMORIM, 2000). The MINI is a short structured diagnostic interview for Diagnostic and Statistical Manual of Mental Disorders-IV (DSM-IV) and International Classification of Diseases (ICD) 10th revision psychiatric disorders. It was validated and translated for use in Brazil. The antisocial personality disorder section includes six questions about conduct disorder before age 15 years and six questions about antisocial behavior after age 15 years. Each has a dichotomous (yes vs. no) response format. Each positive answer was used to create a sum score. In the current study, the six questions about behaviors after age 15 years were used. In addition to asking these questions about their own behavior, mothers were asked to respond to the same questions about the behavior of their partners (MINI was not applied directly to their partners). The final score used in the current study was the sum score for antisocial behavior of the mother and the father, ranging from 0 to 12. The higher the score, the higher the parents’ antisocial behavior.

**Coercive parenting**

Coercive parenting was measured using the Parent and Family Adjustment Scales (PAFAS) (SANDERS *et al.*, 2014) responded to by the mother. This instrument measures parenting practices, quality of the parent–child relationship, parental emotional adjustment and partner and family support in parenting. The 40-item original PAFAS consists of a 28-item Parenting Scale encompassing two domains [parenting practices (17 items) and parent–child relationship (11 items)], and a 12-item Family Adjustment scale encompassing three domains including parental emotional maladjustment (5 items), family relationships (4 items) and parental teamwork (3 items). A validated, reduced, 30-item version of the PAFAS was proposed by SANDERS *et al.* (2014), which includes 18 items on the Parenting Scale: Parental consistency (5 items), Coercive parenting (5 items), Positive encouragement (3 items) and Parent–child relationship (5 items). For both versions, each item is rated on a 4-point scale from “not true of me at all” (0) to “true of me very much” (3). The coercive parenting score ranged from 0 to 15. Higher scores indicate higher levels of coercive parenting.

**Maternal Hostile Attribution Bias**

Maternal hostile attribution bias was measured using the Parental Hostile Attribution Questionnaire (parental HAQ) (HALLIGAN *et al.*, 2007). This instrument examines parental interpretations of ambiguous situations with friends, colleagues and strangers. For each story, mothers answer one hostile interpretation question which assesses their attributions of the other person's intent (on a 6-point Likert-type scale, from “Extremely Unikely” (0) to “Extremely Likely” (5)). The original HAQ contains 6 ambiguous scenarios describing situations, while in this study 5 scenarios were used. We decided to remove one situation which describes a driver in his/her own car, which was deemed inappropriate for most of the cohort mothers. For each ambiguous situation only one hostile interpretation question was used. Each item is scored 0-5, and the mother's responses to the attribution questions are summed across the five stories (with a final score ranging from 0 to 25) (CRICK; DODGE, 1996). The higher the score, the higher the maternal hostile attribution bias.

**Child language**

Language skills were evaluated using both measures of expressive and receptive language (CAPOVILLA; NEGRÃO; DAMÁZIO, 2011). Expressive language was assessed by the University of São Paulo (USP) Expressive Vocabulary Test (TVexp-100) which is a picture-naming test where the interviewer shows a picture book to the child and asks them to name the pictures, i.e. “what is the name of…”. This instrument is composed by 100 items applied directly to the child. Each item is dichotomous (scored 1 for a correct answer and 0 for incorrect) and all are summed to compute the measure's total score (ranging from 0 to 100). Receptive language was assessed by the USP Auditory Vocabulary Test (TVAud-33) which is an instrument composed of 33 items. Each item shows 5 pictures in a line and the interviewer says a word to the child, who is asked to point to the corresponding picture. When the child successfully found the picture, one point is added to the total score (ranging from 0 to 33). In this study, the language variable was scored by the sum of the two aforementioned scores and ranged from 0 to 133. The higher the score, the higher the language development.
